# Gastrointestinal Stromal Tumor: A Clinicopathological Study and Management

**DOI:** 10.7759/cureus.49469

**Published:** 2023-11-27

**Authors:** Aditya Patil, Shriya Haval, Prabhat Nichkaode, Divyansh Dwivedi

**Affiliations:** 1 General Surgery, Dr. DY Patil Vidyapeeth, Pune, IND

**Keywords:** sunitinib, imatinib, gist, prognosis, histopathology, immunohistochemistry

## Abstract

Background and aim

Gastrointestinal stromal tumors (GISTs) account for a major portion of gastrointestinal mesenchymal tumors. The purpose of the current study is to examine the clinicopathological features, management, and therapeutic outcomes of primary GIST in a tertiary care hospital.

Materials and methods

This is a prospective observational analysis. Seventeen patients with GIST have been detected and treated in the Department of Surgery of a tertiary care hospital with an attached medical institution over the last seven years. The clinical presentation, diagnosis method, tumor locations, histopathological results, surgical treatment, and postoperative results were analyzed.

Results

There were six females and 11 males with ages ranging between 35 to 72 years. All the patients had symptoms, with abdominal pain the most prevalent. The most frequent primary site for GIST was the stomach (60-70%), followed by the small intestine (25-30%), the rectum, the esophagus, and the colon (2%). Preoperative diagnosis was made through endoscopy and a contrast-enhanced CT scan. Ninety-two percent of the cases tested positive for CD117. Surgery has been conducted for all 17 patients, with the liver being the most common site of metastasis. Imatinib and sunitinib increased the survival as well as postoperative recurrence rate while decreasing metastasis.

Conclusions

The most general symptom of GIST was abdominal pain. In most instances, it was treated with surgery as well as adjuvant imatinib and sunitinib, and had a favorable prognosis. With increasing size and mitotic activity, the five-year survival rate falls, and the prognosis worsens.

## Introduction

Gastrointestinal stromal tumors (GISTs) are mesenchymal tumors most prevalent in the gastrointestinal (GI) tract, accounting for one percent of all GI neoplasms. Gastrointestinal stromal tumors (GIST) are tyrosine kinase receptor (KIT)-expressing and KIT-signaling driven mesenchymal tumors* *[1]*.* Most tumors previously classified as leiomyosarcoma, leiomyoblastoma, cellular leiomyoma, and leiomyoma are now classified as GISTs [2]. According to theory, mesenchymal stem cells that develop into interstitial cells of Cajal phenotypes are the origin of GISTs. GISTs have a variety of clinical manifestations, which include abdominal pain, acute abdominal mass, chronic GI bleeding, the existence of anorexia, and intestinal obstruction [3]. GISTs in the small bowel (30%) and stomach (60%) are the most common sites, with the esophagus and rectum accounting for the remaining 10% [4]. At present, the expression of CD117 is a sensitive but not entirely specific marker of KIT activation. The gold standard treatment is surgical resection of local disease to achieve complete resection of the tumor while preventing tumor rupture [5]. Complete surgical resection is related to a five-year survival rate in 48 to 65% of patients [6]. The introduction of imatinib and sunitinib has significantly changed the approach towards GIST. It is efficient as a neoadjuvant and adjuvant therapy in metastatic GIST [7].

## Materials and methods

**Figure 1:**
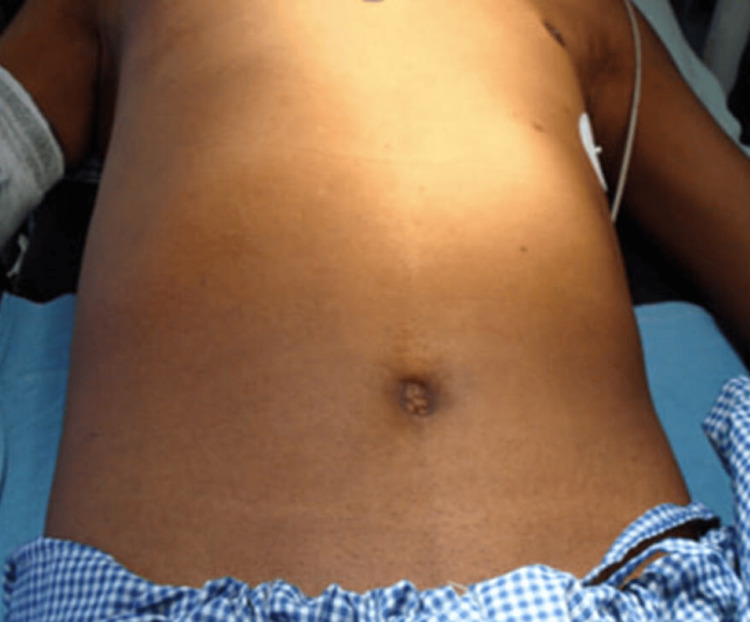
Clinical picture of a patient with a lump in the epigastric region of the abdomen

**Figure 2:**
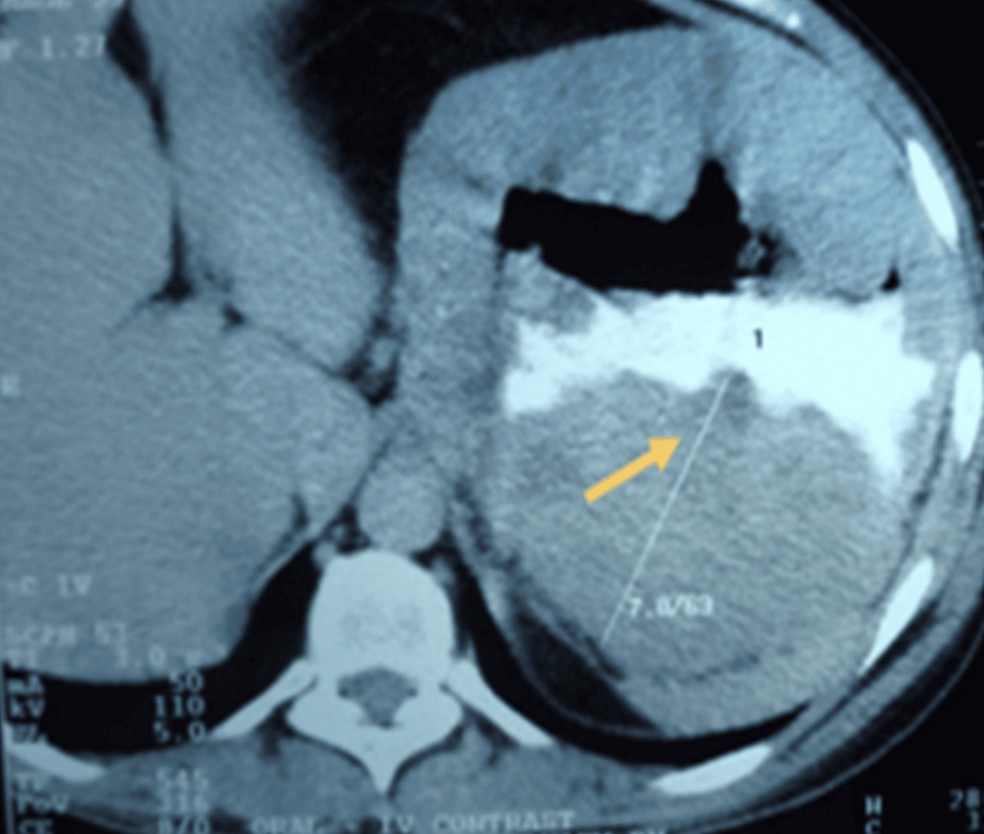
Contrast-enhanced computed tomography of the abdomen and pelvis of the patient with gastrointestinal stromal tumor

Seventeen patients with ages ranging from 35 to 72 years, out of which 11 were males and six were females, came with complaints of la ump in the abdomen (Figure 1) between 2014 and 2022. They were diagnosed with GIST at a tertiary care hospital and were prospectively studied and followed up. The laboratory and standard imaging workup (USG of the abdomen, contrast-enhanced computed tomography (CECT) of the abdomen and pelvis as seen in Figure 2) had been done. Any preoperative biopsies or fine needle aspiration (FNA) from the primary tumor site and PET CT were not performed due to financial constraints. Demographic information, MRI, CT, clinical presentation, lab investigations, and endoscopy results were gathered.

The immunohistochemical profile was created with a S100, CD34, and CD117 panel. Size, mitotic index, rupture, data about the metastatic lymph nodes, and Fletcher's classification [8] were utilized (see Table 1) as prognostic factors. The form of resection was categorized as R0 if there wasn't any microscopic involvement after surgical resection or any residual disease. When there was only microscopic residual illness, it was categorized as R1, and when there was a macroscopic residual disease, it was categorized as R2. In evaluating treatment results, we examined disease-free survival and overall survival alongside survival after imatinib and sunitinib. When used before surgery, treatment with imatinib and sunitinib was defined as neoadjuvant.

**Table 1:** Predicated malignancy potential Source: [8]

Risk of malignancy	Size (cm)	Mitotic (50 hmf)
Very low	less than 2	Less than 5
Low	2 to 5	Less than 5
Intermediate	Less than 5, from 5 to 10	From 6 to 10, less than 5
High	More than 10, any size	Any index, more than 10

## Results

Seventeen patients had open surgical treatment for GIST at a tertiary care hospital. The average age was 55 years, and males had a slightly higher prevalence (71%), with age ranging from 35 to 72 years. Pain in the abdomen was a common complaint, often followed by a lump in the abdomen and generalized weakness. The clinical representation differed depending on the location of the GIST.

For instance, rectal GIST manifests as difficulty defecating and rectal bleeding, whereas gastric GIST presents as a lump in the epigastrium. The stomach was the site of the majority of tumors (eight cases, 47.05%). Other were identified in the small bowel (six cases, 35.29%), ileum (four cases, 23.52%), jejunum (two cases, 11.7%), retroperitoneum (one case, 5.88%), esophagus (one case, 5.88%), and rectum (one case, 5.88%). Tumor sizes ranged from one to 30 cm, with an average diameter of 7 cm. Table 2 displays the clinical features and radio-diagnostic results. 

**Table 2:** Indicates GIST's clinical features and radio-diagnostic results at various sites GIST - gastrointestinal stromal tumor; CECT - contrast-enhanced computed tomography; IVC - inferior vena cava

Sites	Clinical features	Radio diagnostic findings
Stomach	Pain in the abdomen, loss of appetite, generalized weakness, abdomen - rounded, fixed, tender mass in the epigastrium	USG: in the body of the stomach, there is a rounded, vascular mass, well-defined, pedunculated, hetero-echoic mass with calcification. CECT scan: the left lobe of the liver is involved in an exophytic, cavitatory growth that communicates with the stomach posteriorly.
Small bowel	Pain in the lower abdomen, intermittent, colicky, abdomen - tenderness in hypogastrium	USG: 8x5x4 cm hetero-echoic mass originating from the small intestine in the infra umbilical area, with raised vascularity.
Rectum	Rectal bleeding, tenesmus	CECT scan: sigmoid and rectal wall thickening measuring 8 cm long and 3.9 cm thick, most likely neoplastic.
Esophagus	Dysphagia, pain in abdomen	CECT scan: evidence of endophytic growth in the lower third of the esophagus that extends up to the gastroesophageal junction.
Retroperitoneum	Pain in the abdomen, intermittent dull aching, abdomen - mild tenderness in the epigastrium	CECT scan: pancreas was seen compressed by a solid tumor measuring 4x3.5x3 cm into the retroperitoneum between the IVC and abdominal aorta.

**Figure 3:**
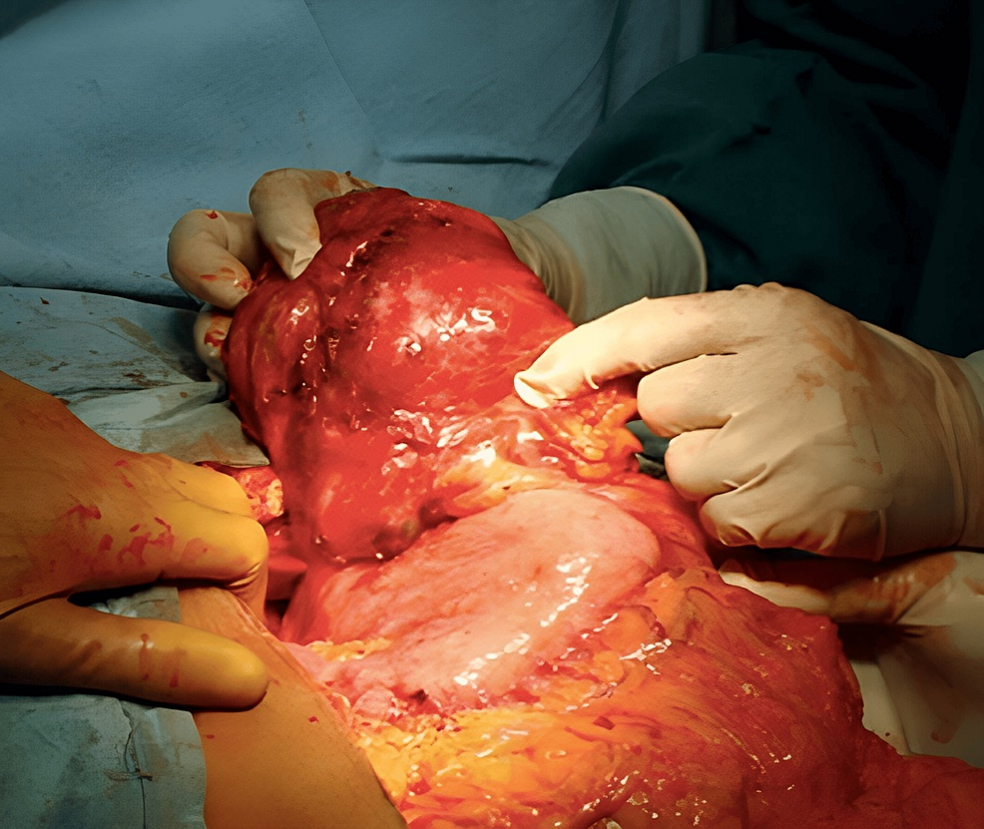
Intraoperative picture of GIST GIST - gastrointestinal stromal tumor

**Figure 4:**
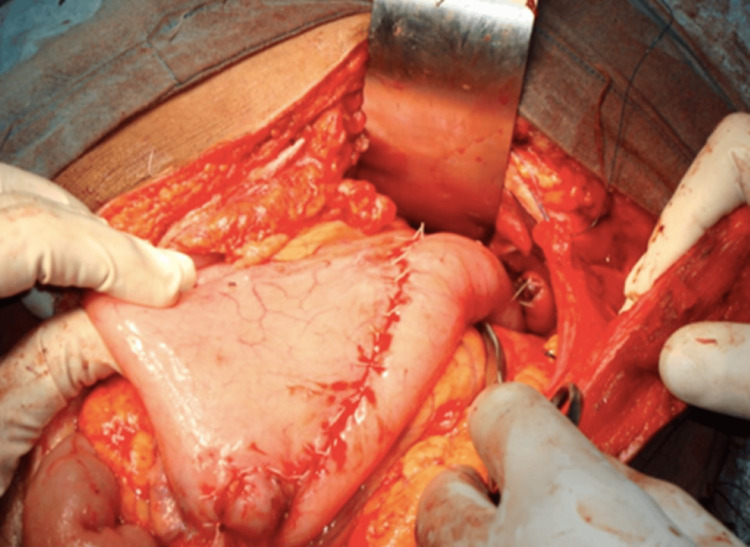
Stomach after wide local excision of GIST GIST - gastrointestinal stromal tumor

The lesion was mainly addressed during exploratory laparotomies performed on all 17 patients. Total tumor excision was possible in all cases apart from two, where the GIST was situated in lesser stomach curvature (Figure 3-4) and had invaded the lesser sac. Other GISTs were located in the retroperitoneum and extended posteriorly up to the abdominal aorta, making them unresectable. The tumor was surgically debulked. R0 resection was achieved in 85.7 percent of the instances, R1 resection in 5.88% (one case), and R2 resection in 5.88% (one case). Three of 17 cases (17.64%) were categorized as high risk, three (17.64%) as intermediate risk, and eight (47.05%) as low risk using Fletcher's classification [8]. In all patients, postoperative periods were uncomplicated. In the last five years of follow-up, eleven of the patients showed no signs of disease recurrence or dissemination. Within two years of surgery, one patient with GIST had recurrence and liver metastasis. He is currently receiving imatinib and sunitinib therapy. Imatinib and sunitinib were administered to all patients with good compliance.

## Discussion

**Figure 5:**
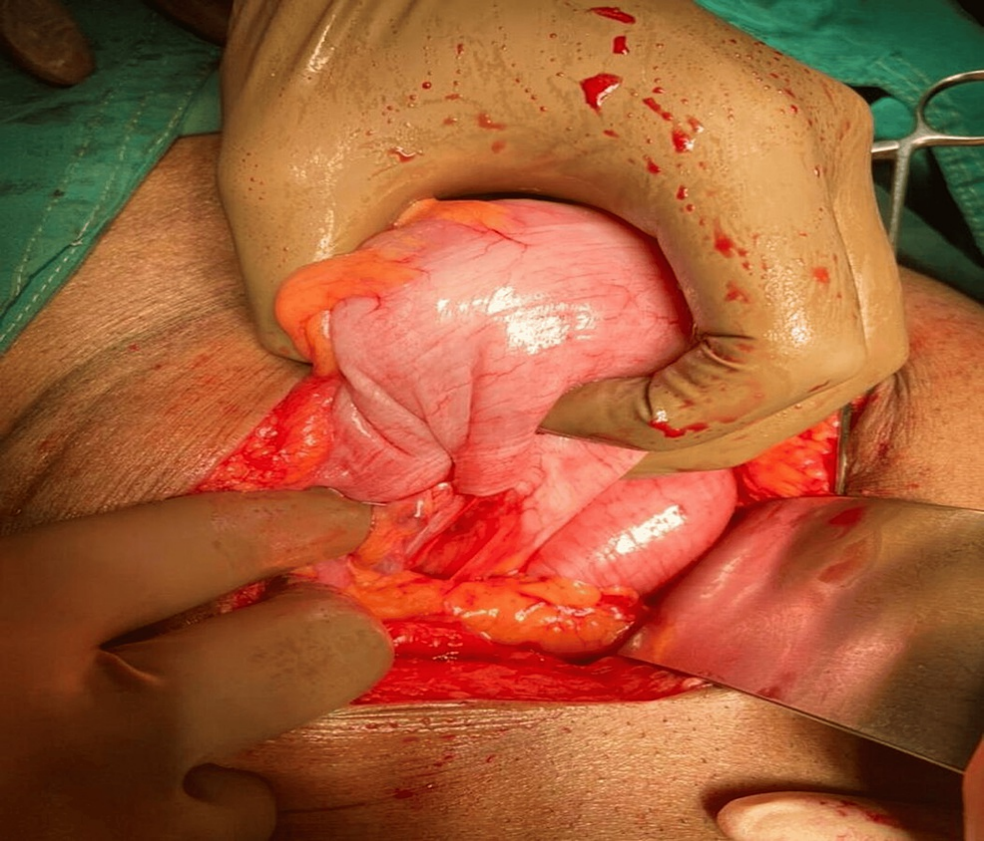
Sigmoid GIST GIST - gastrointestinal stromal tumor

**Figure 6:**
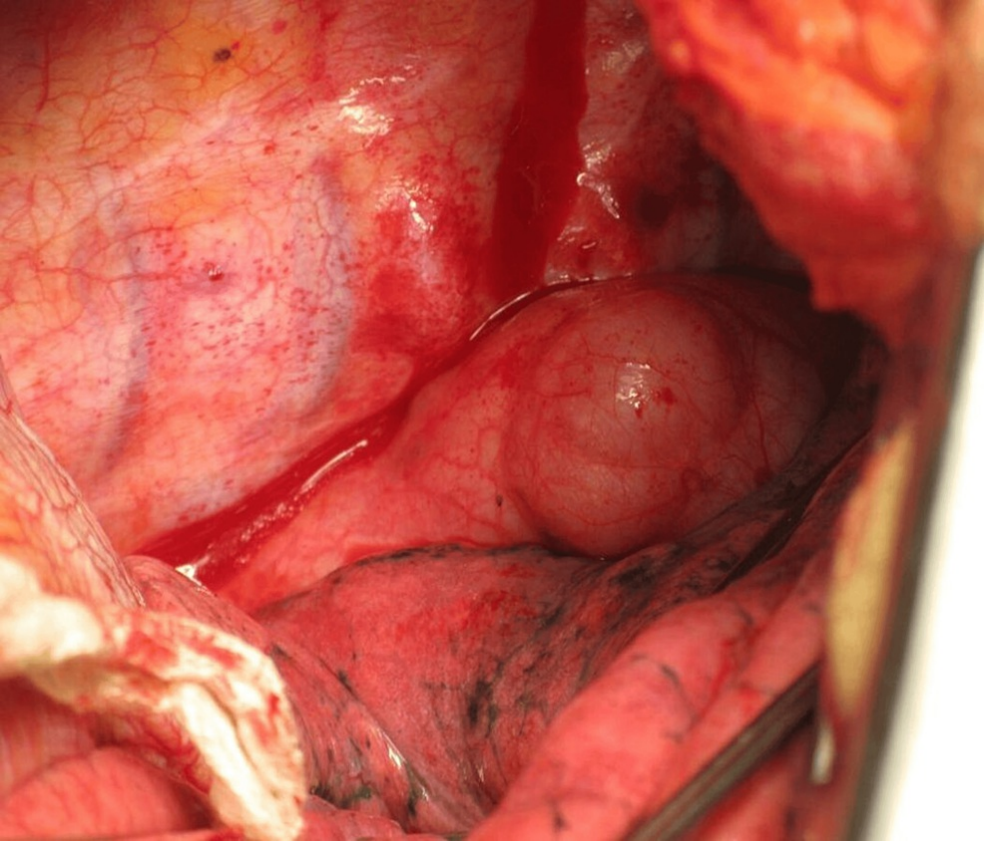
Esophageal GIST GIST - gastrointestinal stromal tumor

As the GIST was identified as a distinct pathological entity with specific features 15-20 years ago, surgical management of GISTs has evolved. Because this type of tumor has no lymphatic spread, lymphadenectomy is unwarranted, so the only oncological standard is to preserve the capsule's integrity and conduct an R0 resection. The most frequent mesenchymal tumors of the GI tract are GISTs, which can develop anywhere along the GI tract. The small intestine (25-35%) and stomach (60-70%) are the most commonly affected, with the colon (see Figure 5) and esophagus (2%; see Figure 6), rectum (5%), and appendix being the least frequently affected.

GISTs possess a variety of clinical manifestations, such as abdominal pain, acute and chronic GI bleeding, the incidence of anorexia, intra-abdominal mass, as well as intestinal obstruction [9]. DeMatteo et al. [10] noted a median age of 58 years, with tumors most commonly found in the stomach (39%), followed by small bowel (32%). Ahmed et al. [11] noted an average age of 64.4 years, with tumors primarily located in the colon (13%) and stomach (52%). Our series had a higher R0 resection rate (85.7%) as compared to others: DeMatteo et al. [10] noted 47%, and Ahmed et al. [11] reported 51%. The difference was explained by the larger spindle cell or epithelioid mesenchymal tumors and morphology, which was somewhat site-dependent. Nevertheless, all these tumors share the KIT (CD117 antigen) expression, a main diagnostic standard [12]. CD117 was positively detected in GISTs; 92.7% of the GIST tumors in the present study stained positively for CD117. It is acknowledged that not all GISTs exhibit CD117 positivity, and CD117-negative tumors frequently include platelet-derived growth factor receptor alpha (PDGFRA) mutations [13]. Table 3 shows operative procedures conducted for immunohistochemistry and histopathology.

**Table 3:** Histopathology and immunohistochemistry of a GIST specimen obtained after site-specific surgical procedures GIST - gastrointestinal stromal tumor; KIT - tyrosine kinase receptor; SMA - superior mesenteric artery

Sites	Operative procedures	Histopathology	Immunohistochemistry
Stomach	Wide local excision, partial gastrectomy, total gastrectomy	There was increased cellularity, mixed cell type, and tumor necrosis	CD117+, CD34+
Small bowel	Excision of the impacted jejunal segment accompanied by an end-to-end jejuno-jejunal anastomosis, end-to-end anastomosis after excision of impacted ileal segment	Spindle cells are dominant, with epitheloid cells as well as a mixed pattern in places; the activity of mitosis is low	KIT, SMA, desmin
Rectum	High anterior resection	A mass of spindle cells that is intramural and extremely cellular	CD117+, CD34+
Retroperitoneum	Debulking of the tumor	Hypocellular tumors composed of spindle cells	CD34+, vimentin
Esophagus	Subtotal esophagectomy with gastroesophageal anastomosis on the neck's left side	A mixture of spindle and epitheloid cell morphology had been identified	KIT, SMA

A pre-operative biopsy is recommended in cases when the tumor seems inoperable on imaging at the time of diagnosis, according to the largest series of 1768 GISTs by Miettinen et al. [14]. The following types of image-guided biopsy are available: endoscopic ultrasound (EUS)-guided biopsy from the primary tumor, EUS-guided FNA, EUS-guided fine needle aspiration biopsy (FNAB), and percutaneous biopsy of primary and metastatic tumors. 

**Figure 7:**
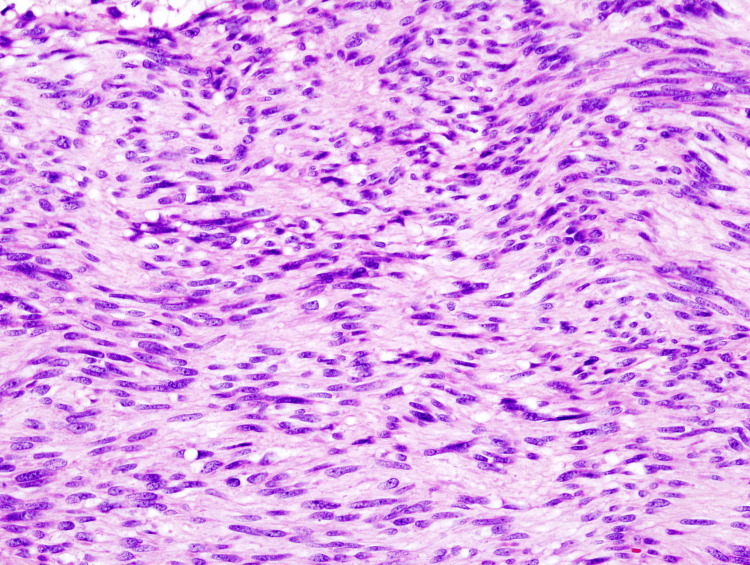
Histopathology of GIST GIST - gastrointestinal stromal tumor

**Figure 8:**
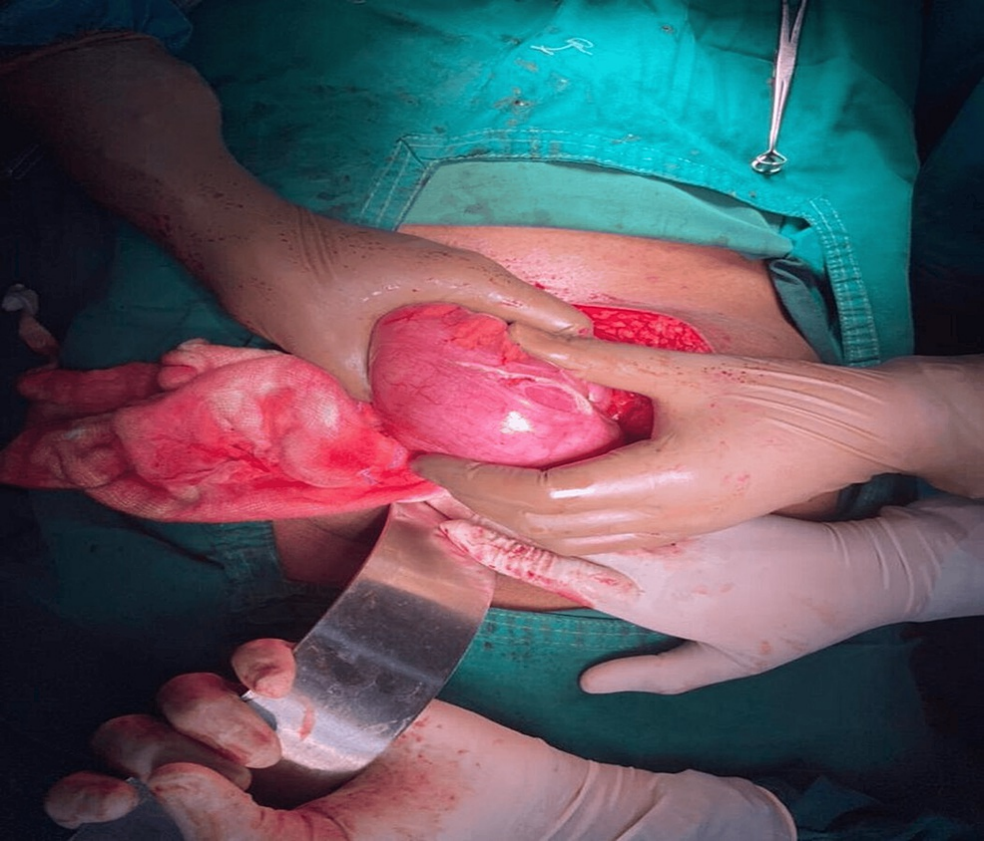
Submucosal GIST GIST - gastrointestinal stromal tumor

Though PET CT is not used for pre-operative diagnosis, its sensitivity is higher than that of CT or MRI. Endoscopy and EUS are used to diagnose tumors with high glucose content [15]. Endoscopy alone or EUS is the preferred method for obtaining tissue for histopathology (Figure 7) in the stomach and colon; on endoscopy, GISTs appear as submucosal masses with regular margins and normal overlying mucosa (Figure 8). EUS may reveal the location of the tumor's origin. A further benefit is the ability to obtain guided tumor tissue for histopathological diagnosis [16]. Ninety-one percent of tumors have been noted to be CD117 positive. The most important method for managing GISTs is complete surgical resection. Aggressive surgical resection, with complete resection, can result in a longer lifespan and might be a potential cure for GIST patients [17]. Survival after starting imatinib and sunitinib was good in terms of treatment results, including overall survival and disease-free survival. Recently, a KIT tyrosine kinase inhibitor called STI-571 (imatinib and sunitinib) has shown potential in the metastatic GISTs treatment. After surgical resection, the five-year survival rate varies from 35-65% [18].

## Conclusions

GIST is a rare mesenchymal tumor of the GI tract. The stomach is the most frequent site, followed by the small bowel, large bowel, and finally esophagus. Ninety-two percent of the cases tested positive for CD117. This condition necessitates a comprehensive approach, which affects patient survival and prognosis. For resectable GISTs, surgery remains the gold standard of care. As described by Fletcher's classification, the most significant determinants for estimating prognosis in the existence of resectable as well as non-metastatic masses that have been appropriately removed by the surgeon continue to be the biological and pathological characteristics of the tumor. Imatinib and sunitinib mesylate, a tyrosine kinase inhibitor, were used in molecular treatment for high-risk or metastatic tumors and non-resectable masses and have proven to better survival in patients.
